# Supplementary material for: Task interruptions from the perspective of work functions: The development of an observational tool applied to inpatient hospital care in France The Team’IT tool
Source: PLoS One. 2023 Mar 9;18(3):e0282721. doi: 10.1371/journal.pone.0282721 (PMC9997927; doi:10.1371/journal.pone.0282721)
Supplement: S1 File — (DOCX) [file pone.0282721.s001.docx]

Supplementary Material S1:

The research team extended this pre-experimental version by adding another 11 items.

Two of these new items were linked to the original item which asked about the duration of the request, and specified its start and end times (A). Two others related to the characteristics of the interrupted task (A), namely whether it was planned or unplanned, and its classification in the patient management program (PMP) (admission/ prescription-development of the care program/ care program delivery/ preparation for discharge/ discharge). Another supplemented the initial question about workload, and asked about the intensity of the activity (low/ medium/ high) being carried out by the interruptee at the time of the request (A). The latter three items were also added to guide B, which targeted the activity of the interrupter. Another item was added to guide A; this asked about the seniority of the professional observed. Finally, two other items were added to guide B; the first described the requested task, and the second asked the interrupter how the system could be reorganized. One item was included in both guides (duration of the request) and was only retained for guide A. In total, 19 items targeted the interruptee, and 16 targeted the interrupter.

The research team retained the response modes proposed by McCurdie *et al*. for the item about how the interruptee responded to the request (ignored the question/ refused to answer/ answered later/ answered while continuing to work/ paid attention to the answer/ other), and the item that categorizes interrupted and requested tasks with respect to the four work functions (unit resource coordination, care coordination, patient care planning, patient care delivery). The item regarding how the request occurred (in person/ telephone/ other) was specified in more detail (in person/ telephone/ medical alarm/ self-interrupted/ other). The classification of the interruptee/ interrupter took into account the principles governing the functioning and environment of team interactions described by Salas *et al*. [28]. Response modes were therefore: administrative; logistical and technical; medical; medicotechnical and psychosocial; paramedical; management; patient (or their entourage); other.
